# Comparative Transcriptome Analysis Reveals Differential Gene Expression in Resistant and Susceptible Watermelon Varieties in Response to *Meloidogyne incognita*

**DOI:** 10.3390/life12071003

**Published:** 2022-07-06

**Authors:** Yingchun Zhu, Gaopeng Yuan, Renzong Zhao, Guolin An, Weihua Li, Wenjing Si, Junpu Liu, Dexi Sun

**Affiliations:** Zhengzhou Fruit Research Institute, Chinese Academy of Agricultural Sciences, Zhengzhou 450009, China; zhuyingchun@caas.cn (Y.Z.); yuangaopeng@caas.cn (G.Y.); zhaorenzong@caas.cn (R.Z.); anguolin@caas.cn (G.A.); liweihua@caas.cn (W.L.); siwenjingsmile@sina.cn (W.S.)

**Keywords:** watermelon, resistant, susceptible, comparative transcriptome, *Meloidogyne incognita*, molecular mechanism

## Abstract

*M. incognita* is a major parasitic plant disease in watermelon production, causing serious economic losses. Although there are many studies on root-knot nematode, the resistance mechanism is still unclear. In this study, in order to fully understand the mechanism of watermelon resistance to root-knot nematode, the relatively strongly resistant ‘Hongzi watermelon’ variety and the susceptible ‘M16’ watermelon variety were used as materials, combined with RNA sequencing (RNA-seq), to analyze the expression abundance of resistant and susceptible varieties at 0, 2, 8 and 15 days post-infection (DPI) by *M. incognita.* The number of differentially expressed genes (DEGs) in the four comparison groups (A0_B0, A1_B1, A2_B2 and A3_B3) was 3645, 2306, 4449 and 2362, respectively, and there were 835 shared DEGs among them. GO annotation and KEGG pathway enrichment analysis showed that 835 DEGs were mainly involved in phenylpropane biosynthesis and carbon metabolism. Furthermore, lignin-biosynthesis-related genes (*4CL* (4-coumaric acid-CoA ligase), *C3H* (coumaric acid 3-hydroxylase), *CSE* (caffeoyl shikimate esterase), *COMT* (caffeic acid-O-methyltransferase), *CCR* (cinnamyl CoA reductase) and *PRX* (peroxidase)), defense-related proteins (UDP-glucoronosyl/UDP-glucosyl transferase, UGT84A13; salicylic acid binding protein, SABP2) and some transcription factors (TFs) were highlighted, which may be potential candidate genes for further analysis in the infection process of *M. incognita*. These results suggest that watermelon can achieve resistance to *M. incognita* by increasing the content of lignin and phenols in root or improving ROS level. These RNA-seq data provide new knowledge for future functional studies and will be helpful to further elucidate the molecular mechanism of resistance to *M. incognita* in watermelon.

## 1. Introduction

Watermelon (*Citrullus lanatus*) is an important horticultural economic crop and is popular among consumers. China is the world’s largest producer and consumer of watermelons. However, with the expansion of watermelon planting area and continuous planting of single varieties, the watermelon growth process is easily affected by pathogens, lack of mineral elements and adverse environmental factors, causing serious economic losses to the actual production. What is more, root-knot nematode (*Meloidogyne* spp.) is becoming more and more frequent and serious [1,2], which is also causing serious harm to watermelon production, resulting in a cut to production of about 30% [3]. Root-knot nematode is one of the soil-borne diseases that seriously damage crops. It has a wide range of hosts and can infect more than 3000 kinds of plants, including food crops, vegetables, fruits, etc. Its damage has exceeded bacteria and viruses, becoming the second largest disease next to fungi. In 2015, the losses caused by plant-pathogenic nematodes in global agriculture reached USD 157 billion [4], among which root-knot nematodes are the most serious. In China, the annual loss caused by root-knot nematode damage is more than RMB 5 billion [4], which has become an important problem restricting the healthy development of China’s agricultural industry.

There are four main kinds of root-knot nematodes that damage horticultural crops: *M. incognita*, *M. arenaria*, *M. hapla* and *M. javanica*, and watermelon is susceptible to all of them [5,6]. According to the survey of the International Meloidogyne Project (IMP), *M. incognita* is the most serious in the world, accounting for about 52% of the collected samples (of which race 1 accounts for 72%). The distribution of root-knot nematodes in China has obvious regional characteristics, among the crops in the protected areas of North China, *M. incognita* is the most prevalent, and the most harmful to the crops. In plants, root-knot nematode can infect the whole growth and development period, mainly damaging the root of the plant and resulting in a limitation of root development, taproom and lateral root deformity and the formation of beaded or chicken-foot root knot [7]. At the beginning of nematode infestation, the roots become white and turn light or dark brown at the later stage, with roughed surface and rotting in severe cases, and the tiny and milky white nematodes can be observed when dissecting the inner part of the root knot. At the initial stage of root-knot nematode infection, there was little effect on the plant, but with the extension of infection, the volume and number of root-knot nematodes increased gradually, resulting in the whole root system becoming nodular [8]. The root activity is significantly affected by the internal nutrient absorption and feeding activity of root-knot nematodes, resulting in yellowing and shrinking of shoots and leaves on the ground and the withering and yellowing of watermelon tendrils, which is similar to a lack of water and fertilizer, finally resulting in the falling of flowers and fruit and poor fruit bearing. In addition, plants damaged by root-knot nematodes are equally vulnerable to other diseases and insect pests, such as *Fusarium wilt*, mosaic virus disease, etc. [9]. Various physical methods were used to control root-knot nematodes, such as heat treatment (high temperature tightly greenhouse, steam treatment), ray or ultrasonic treatment and flooding measures [10], which had adverse effects on the nematodes and inhibited or killed the nematodes. However, although physical measures are effective in controlling root-knot nematodes, they are time-consuming and ineffective for deep-seated active nematodes, so nematodes are difficult to be completely controlled. In addition, the use of various physical control measures is limited due to the influence of different regions, climates and temperatures. At present, chemical control is still the principal method to control root-knot nematodes in agricultural production [11]. However, some chemical agents kill many beneficial microorganisms in the soil while killing harmful nematodes, thus destroying the soil microecology and threatening the environment, human beings and livestock, which is not conducive to the sustainable development of agriculture. Therefore, there is an urgent need for green and efficient control measures to root-knot nematodes.

Planting resistant varieties is one of the more economical, effective and safe control methods. Therefore, it is of great significance and practical value for the control of root-knot nematode by screening resistant germplasm resources and deeply analyzing the resistance mechanism of watermelon. However, in *Cucurbitaceae* crops, there are no directly applicable cultivars for root-knot nematode resistance, but some resistant materials of different degrees have also been found. Thies et al. [12] identified 265 watermelon germplasms by inoculating race 3 of *M. incognita* and found that the overall resistance of watermelon for feeding was medium resistance, among which PI 482303 was the strongest. Shen et al. [13] obtained 5 watermelon germplasms resistant to *M. incognita* from 25 local varieties. In the rootstock grafting experiment, Thies et al. [14] found that forage watermelons (*C. lanatus* var. *citroides*) could be used as resistant rootstock. Deng et al. [15] also identified a germplasm with better resistance than PI 482303—‘Hongzi watermelon’. Punithaveni et al. [16] showed that the resistance of medicinal watermelon to *M. incognita* was better than that of zucchini. These results suggested that the resistance of different watermelon varieties to *M. incognita* is different, and the difference may be regulated by resistance genes. Therefore, it is necessary to carry out transcriptomic research.

Transcriptomics is the study of gene transcription and transcriptional regulation in cells by sequencing a set of different numbers of transcripts in cells with specific physiological status at the overall level, so as to interpret the biological mechanism of plant stress resistance and disease resistance. With the rapid development of high-throughput sequencing technology, RNA-seq technology has been widely used [17,18]. In *Arabidopsis*, rice, tobacco and other model organisms, the interaction between the host and the nematode, the metabolic pathway and the giant cell formation mechanism have been studied in detail by RNA-seq. Kyndt et al. [19] revealed the different interaction mechanisms between rice and root-knot nematode through RNA-seq technology. Olga et al. [20] studied the genome-wide expression analysis of the interaction between alfalfa-resistant varieties and *M. incognita*, revealed the difference of gene expression level of resistant varieties when infected by nematodes, and obtained candidate genes related to *M. incognita* resistance through functional prediction and classification. In *Cucumis metuliferus*, Xue et al. [21] found that three metabolic pathways (jasmonic acid metabolism, phenylalanine biosynthesis and phenylalanine metabolism) may be involved in the nematode resistance of *C. metuliferus* by RNA-seq analysis, and AP2, MYB and WRKY TFs were also involved in nematode resistance. Li [22] used the Illumina HiSeq 2500 high-throughput sequencing platform to generate the whole genome expression profile of *C. metuliferus* and preliminarily revealed that transcription factor AP2 may be closely related to the *M. incognita* resistance gene in *C. metuliferus*. Ling et al. [23] used RNA-seq to reveal that cytoskeleton-related genes are key regulatory genes of resistance to *M. incognita*. Li et al. [24], through RNA-seq technology, analyzed the resistant soybean variety ‘Huipizhi Heidou’ infected by soybean cyst nematode and obtained 740, 1165 and 2925 DEGs at DPI5, DPI 10 and DPI15, respectively. The upregulated genes were mainly enriched in defense responses, hormone-mediated signaling processes and responses to stress. In this study, resistant variety ‘Hongzi watermelon’ and the susceptible variety ‘M16’ were used as experimental materials. Firstly, we identified their resistance to *M. incognita* and then observed the development of root knot nematode at different stages after infection, revealing the infection characteristics of *M. incognita* to resistant and susceptible watermelon varieties. Finally, RNA-seq technology was used to analyze the root transcript’s abundance of resistant and susceptible watermelon varieties after *M. incognita* infection and to identify resistance-related DEGs, which provided the theoretical basis for the breeding of watermelon resistant to *M. incognita.*

## 2. Materials and Methods

### 2.1. Plant and Nematodes Material, Growth Conditions

‘Hongzi watermelon’ and ‘M16’ were used as experimental materials. ‘Hongzi watermelon’ was one of the seed watermelon (*C. lanatus* var. *citroides*) (developed root system, leaf length is about 25 cm, the internode length is about 10 cm, yellow peel, seed length is about 1 cm), and the preliminary identification results showed that it was resistant to *M. incognita*; the pure inbred line ‘M16’ is a commonly cultivated watermelon (*C. lanatus* var. Lanatus (developed root system, leaf length is about 15 cm, the internode length is about 10 cm, green peel, seed length is about 0.3 cm), and the preliminary identification results showed that it was susceptible to *M. incognita*. The above two materials were preserved in our laboratory. Race 1 (which was the most widely distributed species in China) *M. incognita* was provided by Professor Li Hongmei, College of Plant Protection, Nanjing Agricultural University, and was preserved and propagated on the susceptible tomato variety ‘Xifen 902’ in the greenhouse.

### 2.2. Extraction, Hatching and Collection of Root-Knot Nematode Eggs

According to the method of Wang [25], the roots of tomato seriously damaged by *M. incognita* were soaked in water, and the substrate and soil on the root system were washed. The roots were cut into 1 cm pieces and put into a blender for treatment with an appropriate amount of water homogenization. The homogenate passed three times though screens of 20 (3×), 60 (3×), 200 (3×) and 500 mesh (3×), and this step was repeated three times. The clean eggs were collected in a centrifuge tube, the impurities were removed and eggs were concentrated with 36% sucrose flotation. Finally, the eggs were placed on top of a 500 μm nylon membrane and then incubated in a dark incubator at 28 °C for 2 days. Two days later, newly hatched second instar nematodes were collected in petri dishes.

### 2.3. Inoculation and Developmental Status Analysis of M. incognita

The root-knot nematodes were inoculated when the watermelon seedlings grew 4–5 true leaves. In detail, ~1 cm away from the base of the rhizome, a 2 cm deep hole was evenly drilled with a glass rod, and 2 mL second instar larva nematodes suspension was gently injected into the small hole with a pipette gun. The inoculation number was 2000 per plant. A container inoculated with nematodes was cultured in a plastic greenhouse, and the roots were taken regularly for detection.

At 2, 4, 6, 8, 11, 15, 20 and 30 DPI of *M. incognita*, 10 plants of ‘Hongzi watermelon’ (short for A) and ‘M16’ (short for B) with the same growth were collected to count the number of root knots. Twenty root nodules were randomly selected at each period, and the diameter of root nodules was measured under a microscope. Then, the roots were stained with acid fuchsin method, and the development of nematode was observed and compared under electron microscope, measuring the width of root-knot nematode. Each experiment was repeated three times.

### 2.4. Total RNA Extraction and CDNA Library Preparation

According to the above results, root tissues of ‘Hongzi watermelon’ and ‘M16’ seedlings with consistent growth were selected for sampling at 0, 2, 8 and 15 DPI. The ‘Hongzi watermelon’ samples were marked as A0, A1, A2 and A3, and the ‘M16’ samples were marked as B0, B1, B2 and B3. There were three biological repeats in each period for a total of twenty-four samples. The 0 d samples were set as the control, and the other samples were set as treatments. After the root of the sample was washed clean, the residual water on the surface was dried with filter paper, and then frozen in liquid nitrogen immediately and stored in −80 °C refrigerator for RNA extraction.

### 2.5. RNA-Seq Data Sequencing, Assembly and Annotation

Total RNA was extracted using the RNA Prep Pure Plant Kit (TIANGEN, Beijing, China). The integrity of the extracted RNA was determined by 1% agarose gel electrophoresis, and the quantity and quality of the RNA samples were determined using Agilent 2100 RNA Nano 6000 Assay Kit (Agilent Technologies, Santa Clara, CA, USA). RNA samples were sent to Beijing Annoroad Biotech for library construction and Illumina sequencing. Briefly, the RNA was reverse-transcribed into cDNA, and the cDNA fragments were purified using Agilent 2100 RNA Nano 6000 Assay Kit. Then, the cDNA was subjected to terminal repair, ligation and PCR amplification to obtain a cDNA library. Finally, the constructed cDNA library was sequenced using the Illumina platform, and the sequencing strategy was PE150.

The clean data were obtained from raw data by removing the adaptor-polluted reads, the low-quality reads and reads with a number of N bases (N base means any base, which means that the software cannot tell which base it is, so it has to be removed because the sequencing is so bad), accounting for more than 5% of the samples. Then, statistical analyses were carried out on the clean data for their quantity and quality, including Q20, Q30, data quantity and base content statistics.

The watermelon genome (cv. 97103) version 2 from the Cucurbit Genomics Database [26] was used as the reference genome. Bowtie/Bowtie2 was used for building the genome index, and clean data were mapped to the reference genome using TopHat v2.0.12 [27]. Then, the StringTie software was used to splice the mapped reads based on the selected reference genome sequence and compared to the original genome annotation information. Fragments Count for each gene in each sample was counted by HTSeq v0.6.0, and FPKM (Fragments Per Kilobase Per Million Mapped Fragments) were calculated to estimate the expression level of genes in each sample. Genes with q ≤ 0.05 and |log2_ratio| ≥ 1 were identified as DEGs. In this study, the DEGs were identified according to their expression levels in different samples, and further functional annotation and enrichment analysis were carried out based on the comparison results.

Gene annotation and functional assignments were carried out based on the Nr, Swiss-prot, KEGG and GO databases. GO and KEGG annotation results, official classification and functional classification were performed for the DEGs. KEGG pathway enrichment of DEGs was implemented by the hypergeometric test, in which *p*-value is calculated and adjusted as q-value, and the data background is genes in the whole genome. GO terms or KEGG terms with q < 0.05 were considered to be significantly enriched.

### 2.6. Quantitative Real-Time (qRT)-PCR Validation Analysis of DEGs

Total RNA was extracted with the Plant RNA Kit (Huayueyang Biotechnology Co., Ltd., Beijing, China). A total of 1.0 μg of RNA was used for synthesizing cDNA using a PrimeScript RT regent Kit with gDNA Eraser (TaKaRa, Tokyo, Japan) according to the manufacturer’s protocol. All primers were synthesized by SunYa (Zhengzhou, China). qRT-PCR was performed on the Light Cycler480 Real-Time System (Bio-Rad Laboratories, Hercules, CA, USA) with the following steps [28]: 45 cycles of 95 °C for 5 min, 95 °C for 10 s, 58 °C for 10 s and 72 °C for 10 s, followed by a melting curve analysis. Each reaction mix contained 1.0 μL previously diluted cDNA (1:5), 10.0 μL TB Green Premix Ex TaqTM II (Tli RNaseH Plus) (TaKaRa) and 10.0 mM of each primer, for filling a final volume of 20 μL using 7 μL RNase-free water. At least three biological replicates were performed for each PCR amplification. Online software (https://www.ncbi.nlm.nih.gov/tools/primer-blast/index.cgi?LINK_LOC=BlastHome, accessed on 13 April 2022) was used to design the primers of DEGs and synthesized by Sangon (Shanghai, China). All of the primers of DEGs were listed in Table S1. We used *Actin* as the reference gene, and the primer sequences were (Forward primer: 5′-GAACTTGGCACCTGTCCTGT-3′ and reverse primer: 5′-GAACAGTGCAACAGCCTCAA-3′). Relative gene expression values were calculated using the 2^−ΔΔCt^ method [29].

Excel 2016 software was used for data analysis, SPSS 18.0 software (SPSS, Inc., Chicago, IL, USA) was used to sort out the data for one-way ANOVA statistical analysis and the significant difference was defined as *p* < 0.05 (n = 3).

## 3. Results

### 3.1. Analysis of Root-Knot Development

**Figure 1:**
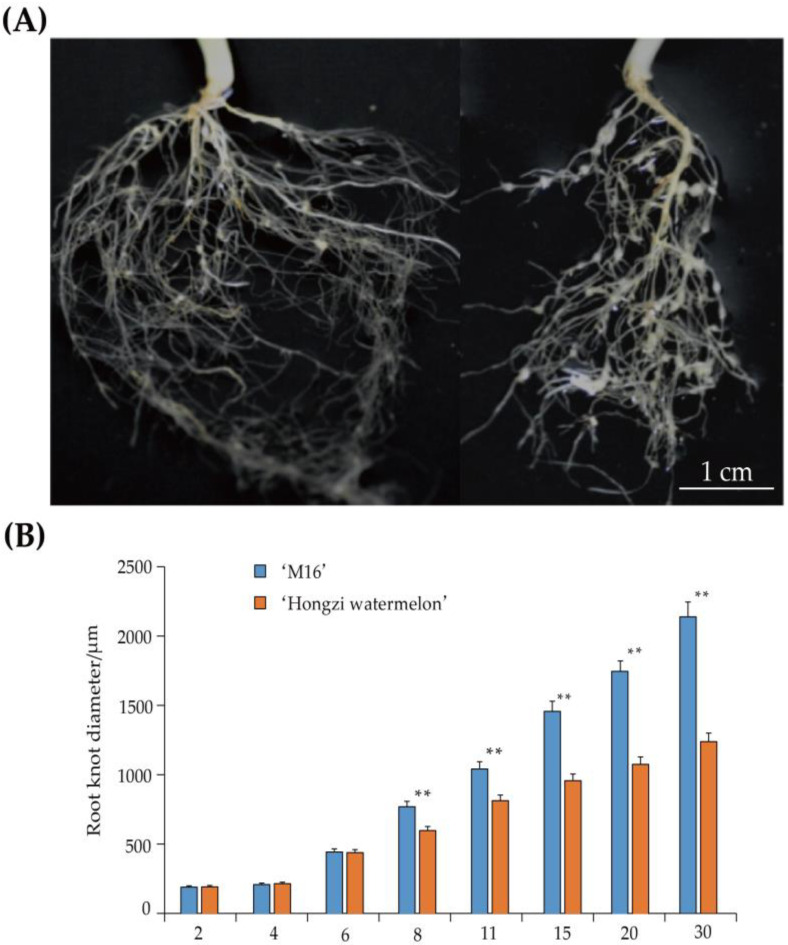
The development of root knot. (**A**) The symptoms of ‘Hongzi watermelon’ (left) and ‘M16’ (right) after *M. incognita* infection at 30 days; (**B**) comparison of root knot diameters of ‘Hongzi watermelon’ and ‘M16’. The ** indicates significant difference at 0.01 level.

In order to understand the root-knot development of resistant and susceptible watermelon varieties infected by *M. incognita*, we analyzed the number of root-knots in ‘Hongzi watermelon’ and ‘M16’ at DPI30, and the results showed that their root-knot numbers were significantly different (Figure 1A). The number of root-knots in ‘M16’ was 58.4, which was significantly higher than that of ‘Hongzi watermelon’ (18.8). In addition, only a few small root nodules were observed in the roots of ‘Hongzi watermelon’, indicating that it is resistant to *M. incognita* while ‘M16’ is susceptible to *M. incognita*.

Then, we investigated the developmental state of the root-knots at DPI2, DPI4, DPI6, DPI8, DPI11, DPI15, DPI20 and DPI30 (Figure 1B). At DPI2 and DPI4, tiny root nodules were observed in the roots of both resistant and susceptible varieties, but there were no significant differences in the size of the root nodules. At DPI6, the roots of resistant and susceptible cultivars began to expand, and the root nodules increased sharply, but there continued to be no significant difference. When it came to DPI8, DPI11, DPI15, DPI20 and DPI30, the root node diameters of the resistant variety were all significantly different (*p* < 0.01). Especially for DPI30, the average diameter of ‘Hongzi watermelon’ was only 1.20 mm, while the root node diameter of ‘M16’ was 2.08 mm.

### 3.2. Analysis of Root-Knot Nematode Development

**Figure 2:**
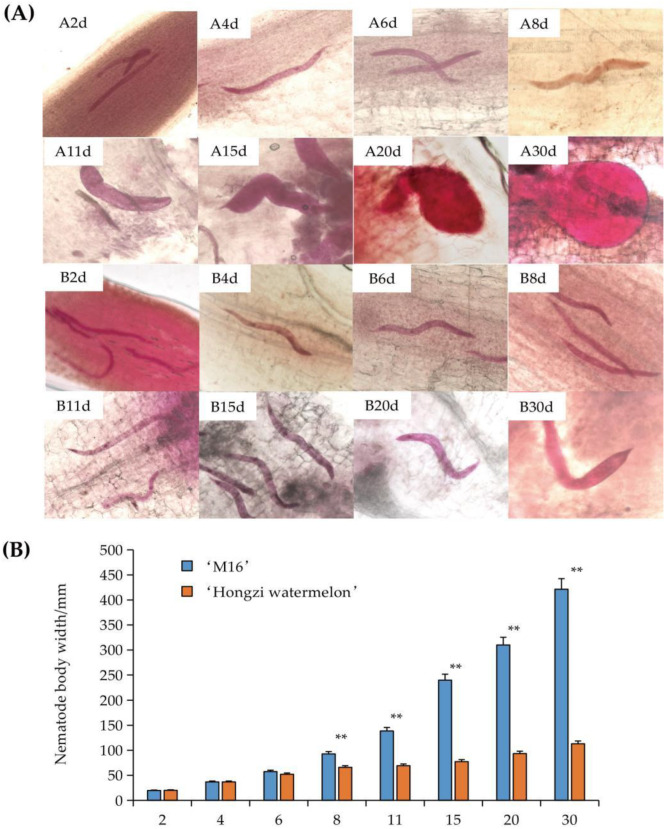
Analysis of root-knot nematode development. (**A**) The development comparison of ‘Hongzi watermelon’ and ‘M16’ at different days post-infection. (**A**,**B**) represents ‘M16’, ‘Hongzi watermelon’, respectively; (**B**) comparison of nematode body width in ‘Hongzi watermelon’ and ‘M1 6’. The data followed (**A**,**B**) represent the DPI of 2 d, 4 d, 6 d, 8 d, 11 d, 15 d, 20 d and 30 d. The ** indicates significant difference at 0.01 level.

We also analyzed the development of root-knot nematode in resistant and susceptible varieties by acid fuchsin staining. The results showed that with the extension of infection time, the root-knot nematodes began to expand, and the development rate of the nematodes in the ‘M16’ roots were significantly higher than those of ‘Hongzi watermelon’ (Figure 2A).

At DPI4 and DPI6, the body widths of the nematodes in the resistant and susceptible varieties were significantly increased compared to DPI2, but there was no significant difference between the two varieties. At DPI8, the nematode in the root of ‘M16’ showed the tendency of accelerating expansion, and the increase in body width was much larger than that of ‘Hongzi watermelon’; the difference reached an extremely significant level. The nematode body width of ‘M16’ was 92.8 μm, which was significantly larger than that of ‘Hongzi watermelon’ (66.0 μm) (Figure 2B). At DPI15, the nematode in the ‘M16’ root was mature, and there were already eggs in the body, where a small number of eggs were found to be produced. At DPI30, the nematode presented a fully matured pear shape, at which time the difference between the two varieties reached the maximum, the body widths was 421.4 μm and 113.0 μm, respectively (Figure 2A,B). At this time, nematodes of ‘Hongzi watermelon’ only entered J3 and J4 stages, and only a few of them developed into adults. These results indicated that the growth and development of *M. incognita* in ‘Hongzi watermelon’ were inhibited.

### 3.3. Transcriptome Sequencing and Read Mapping to Watermelon Genome

In order to fully understand the gene expression changes in resistant and susceptible watermelon varieties in response to *M. incognita* infection, eight cDNA libraries were constructed from the root tissue of seedlings at 0, 2, 8 and 15 DPI, namely, A0, A1, A2 and A3 for the resistant variety and B0, B1, B2 and B3 for the susceptible variety. Illumina HiSeq 2000 platform was used for RNA sequencing. A total of 156.42 GB of clean data was obtained, the clean data of each sample reached 6.00 GB and the Phred score of the ≥Q30 bases was more than 95.19% for each sample. The GC content was very similar among samples and ranged from 44.28% to 46.43%. The proportion of clean reads that mapped to the watermelon genome of each sample ranged from 88.06% to 96.15% (Table S2). Furthermore, gene expression was analyzed based on the comparison results.

### 3.4. Analysis of DEGs

**Figure 3:**
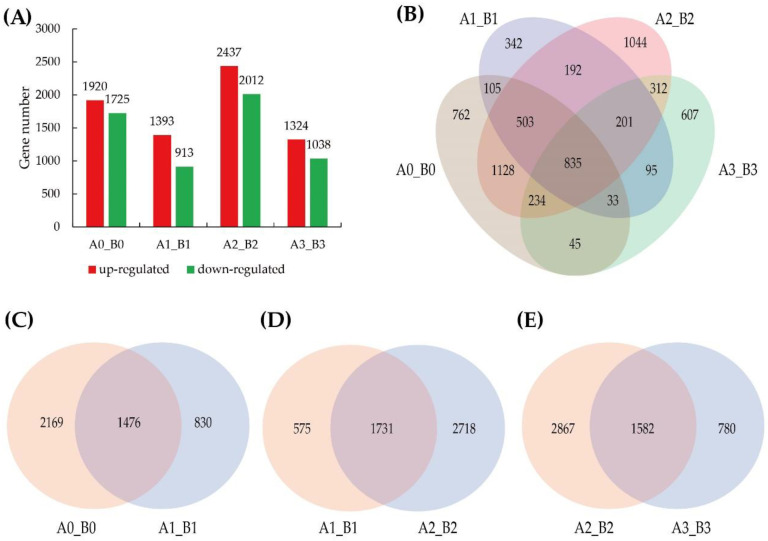
Histogram and Venn diagram of DEGs during *M.* *incognita* infection. (**A**) The number of DEGs at different infection time points; (**B**) The Venn diagram from the four comparison groups, A0_B0, A1_B1, A2_B2, A3_B3; (**C**–**E**) The Venn diagram of DEGs from A0_B0 vs. A1_B1, A1_B1 vs. A2_B2 and A2_B2 vs. A3_B3, respectively.

In this study, a total of 12726 DEGs were identified from four comparison groups, including 3645 DEGs from the A0_B0 group, 2306 DEGs from the A1_B1 group, 4449 DEGs from the A2_B2 group and 2362 DEGs from the A3_B3 group (Figure 3A). Furthermore, we found that 1476 DEGs were shared in A0_B0 vs. A1_B1, 1731 in A1_B1 vs. A2_B2 and 1582 in A2_B2 vs. A3_B3 (Figure 3C–E). Out of these DEGs, 835 DEGs were shared in all four groups, among which 543 up-regulated DEGs and 291 down-regulated DEGs by a Venn diagram (Figure 3B).

### 3.5. Functional Annotation and Classification of the DEGs

**Figure 4:**
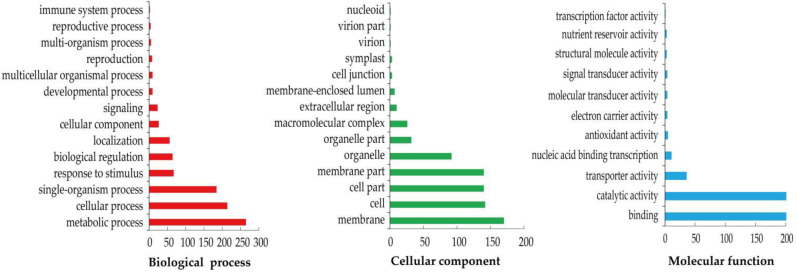
GO classification of 835 shared DEGs during *M.* *incognita* infection. The three main GO categories (biological process, cellular component, and molecular function) were showed. The Y-axis indicated GO-terms, and the X-axis indicated the number of DEGs.

In order to better understand the putative function of the DEGs, GO function annotation and classification analysis were carried out based on the above 835 shared DEGs (Figure 4). These DEGs fell into three categories, including biological process (936), cellular component (768) and molecular function (538). Among these, ‘metabolic process’ (28.31%) and ‘cellular process’ (22.86%) were the most prominent terms in the biological process, while ‘membrane’ (22.14%) and ‘cell’ (18.49%) were the most abundant terms for the cellular component. In addition, for molecular function, ‘catalytic activity’ (47.03%) and ‘binding’ (39.78%) were the most enriched terms. Furthermore, ~9.83% of the DEGs responded to stress-response-related biological process: for example, ‘response to stimulus’, ‘signaling’, and ‘immune system process’ were notably enriched.

**Figure 5:**
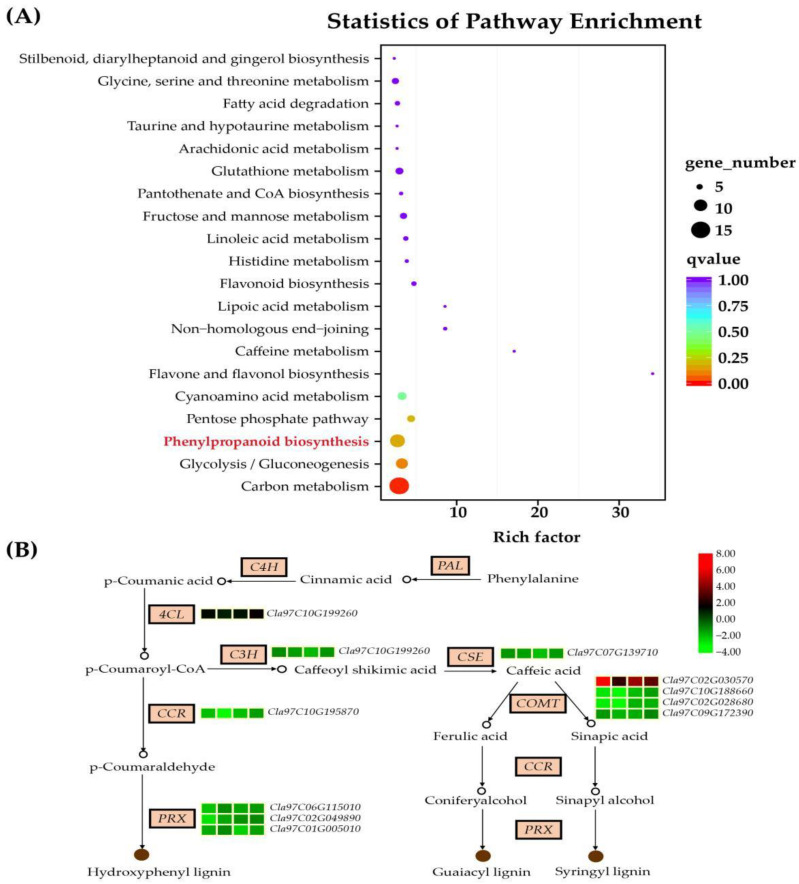
KEGG pathway enrichment analysis of 835 shared DEGs during *M.* *incognita infection*. (**A**) The top 20 pathways; (**B**) Expression level of genes related to lignin biosynthesis pathway. Each row represented four comparison groups (A0_B0, A1_B1, A2_B2 and A3_B3) from left to right. The log2|A/B| was colored using TBtools [30]: red color indicated the DEG was up-regulated, and green color indicated it was down-regulated.

To further identify which biological pathways were significantly different during *M. incognita* infection, we carried out KEGG pathway enrichment analysis for the 835 DEGs. The results showed that 124 DEGs were successfully annotated into 75 KEGG pathways, out of which the pathways with rich factors ranked in the top 20 were selected for further analysis (Figure 5A, Table S3). The most enriched categories were carbon metabolism (14.52%, ko01200) and phenylpropanoid biosynthesis (10.49%, ko00940). In addition, glycolysis/gluconeogenesis (ko00010), cyanoamino acid metabolism (ko00460), pentose phosphate pathway (ko00030), glutathione metabolism (ko00480), fructose and mannose metabolism (ko00051) and glycine, serine and threonine metabolism (ko00260) pathways were also predominantly enriched. Furthermore, one of the main products of this pathway is lignin, which is an important defense against various infections. There was a total of 13 genes involved in the phenylpropanoid biosynthesis pathway, and 11 of them encode enzymes related to lignin biosynthesis (Figure 5B), including one *4CL*, one *C3H*, one *CSE*, four *COMTs*, one *CCR* and three *PRXs*.

### 3.6. DEGs Related to Transcriptional Factors during M. incognita Infection

There were 54 genes encoding transcription TFs among the shared 835 DEGs during *M. incognita* infection (Table S4). According to previous studies, TFs such as AP2, WYKY, MYB, HSF, Zinc finger protein and MADS play important roles in defense against stress [31,32].

**Figure 6:**
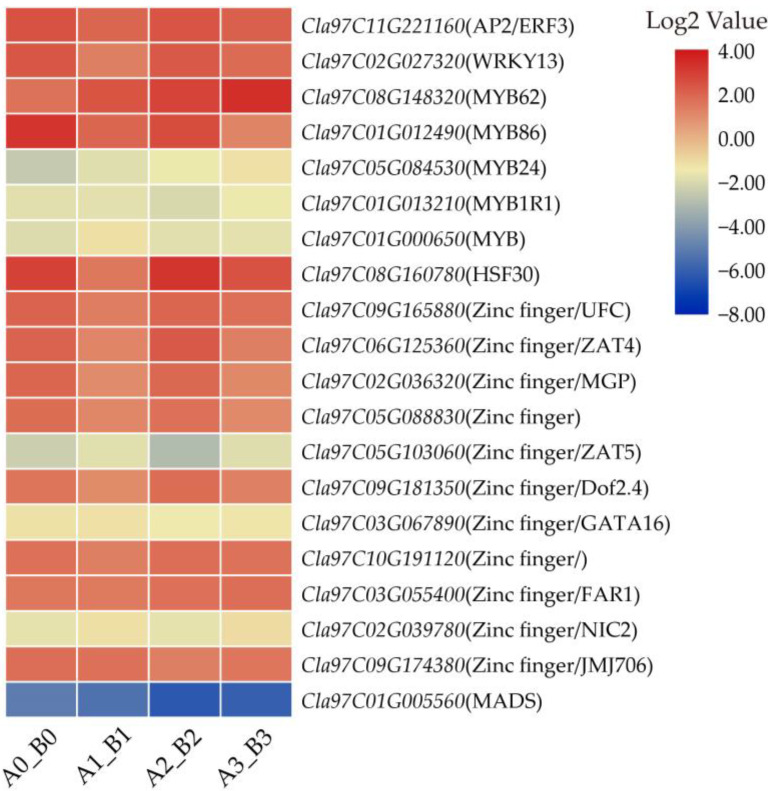
Heatmaps of DEGs encoding transcriptional factors. Each row represented four comparison groups (A0_B0, A1_B1, A2_B2 and A3_B3) from left to right. The log2|A/B| was colored using TBtools, red color indicated the DEG was up-regulated, and blue color indicated it was down-regulated.

In this study, we found that one AP2, one WYKY, five MYBs, one HSF, eleven Zinc finger proteins and one MADS were differentially expressed, and most of them were up-regulated (Figure 6, Table S4). For example, AP2, WRKY, HSF and 8 of the 11 Zinc finger protein genes in our data were significantly up-regulated in the resistant variety. These results indicated that the genes that encode TFs work together to play a role in *M. incognita* resistance.

### 3.7. DEGs Related to Phytohormones

**Figure 7:**
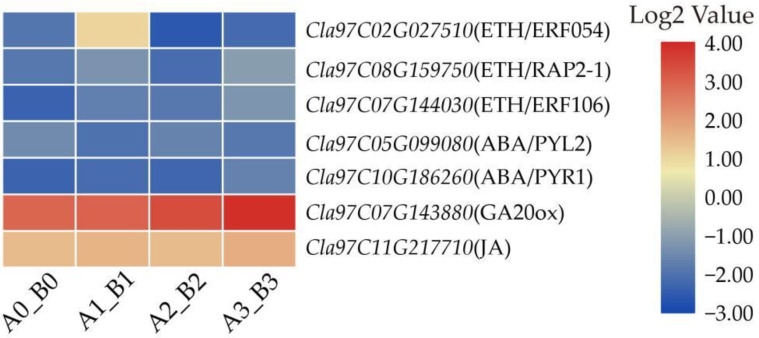
Heatmaps of DEGs related to phytohormones. Each row represented four comparison groups (A0_B0, A1_B1, A2_B2 and A3_B3) from left to right. The log2|A/B| was colored using TBtools: red color indicated the DEG was up-regulated, and blue color indicated it was down-regulated.

Furthermore, DEGs were screened to investigate the role of phytohormone-related genes in *M. incognita* infection, including three ethylene (ETH)-, two abscisic acid (ABA)-, one gibberellin (GA20ox)- and one jasmonic acid (JA)-related genes (Figure 7). In plants, ETH and ABA generally play a role in promoting senescence, interestingly, our data showed that the three ETH-related genes (*Cla97C02G027510*, *Cla97C08G159750*, *Cla97C07G144030*) and two ABA-related genes (*Cla97C05G099080*, *Cla97C10G186260*) were all down-regulated, indicating that the infection of *M. incognita* may promote the senescence of the susceptible variety.

### 3.8. DEGs Related to Defense-Related Proteins

**Figure 8:**
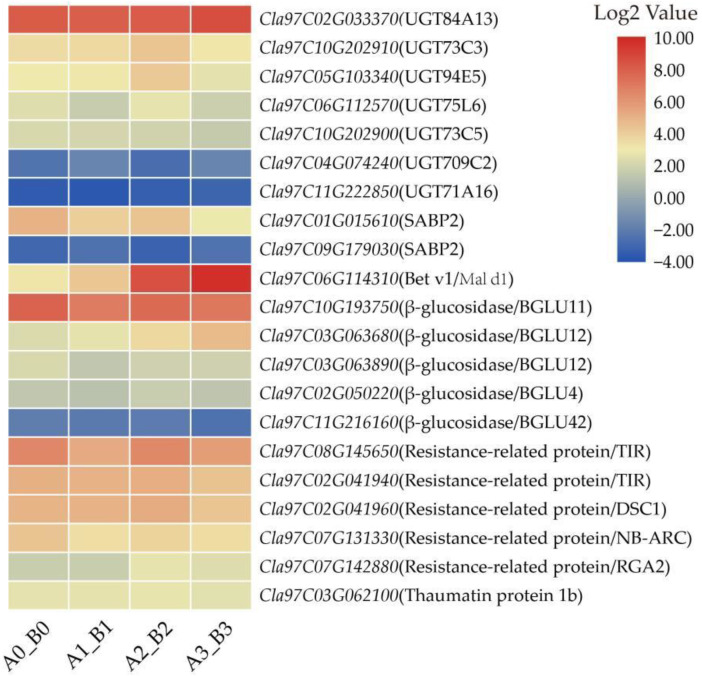
Heatmaps of DEGs related to defense-related proteins. Each row represented four comparison groups (A0_B0, A1_B1, A2_B2 and A3_B3) from left to right. The log2|A/B| was colored using TBtools: red color indicated the DEG was up-regulated, and blue color indicated it was down-regulated.

UDP-glucoronosyl/UDP-glucosyl transferase (UGT) participates in the hypersensitive reaction of plants by synthesizing some resistant substances, such as scopoletin glucoside, scopoletin and betacyanins [33,34,35]. In our data, we identified seven *UGT* genes, and five of them were up-regulated (Figure 8). One up-regulated gene encoding the Bet v1 family protein (Cla97C06G114310) was also identified. Under *M. incognita* infection, pathogenesis-related proteins rapidly accumulate, including five β-glucosidase and five resistance-related proteins, and all of them except *Cla97C11G216160* were up-regulated. Thaumatin protein (Cla97C03G062100) was involved in defense responses regulated by salicylic acid (SA), and the protein was expressed in a pattern similar to the gene that encodes SA binding protein (Cla97C01G015610).

### 3.9. QRT-PCR Validation of Transcriptome Data

**Figure 9:**
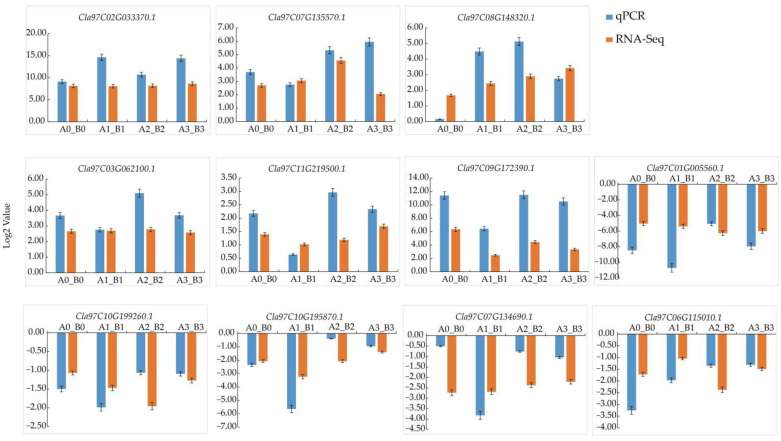
Validation of DEGs by qRT-PCR analysis.

We selected 11 DEGs from the transcriptome data for qRT-PCR analysis in order to verify the accuracy of RNA-seq data. The results showed that the qRT-PCR data were consistent with the expression trend of the RNA-Seq data, implying that the RNA-Seq data obtained by Illumina sequencing have high reliability (Figure 9). The inconsistencies between the data sets may be explained by differences between the two methods.

## 4. Discussion

Breeding and using resistant varieties is the most economical and effective method to control root-knot nematode because agricultural control technology is not easy to achieve and its effect is poor, and chemical control also has the problems of food safety and environmental pollution. Previous studies on the development of *M. incognita* in the roots of *Cucumis metuliferus* (‘CM3’) revealed that the resistance to *M. incognita* was produced by inhibiting its development [36,37], and the resistance was still observed at 35 °C. For example, in the ‘CM3’ roots, the *M. incognita* could not complete its life cycle at DPI28, and there were serious diapause and shrinkage death of nematodes, which revealed the anti-invasion and anti-growth characteristics of ‘CM3’ to the nematode [36]. In the screening and evaluation of watermelon rootstock resources resistant to *M. incognita*, Wang [38] concluded that the resistance of ‘Hongzi watermelon’ was the strongest among all tested materials from the comparison of the disease index, the root knot index, the egg grain index and the nematode reproduction coefficient. In this study, we further confirmed that ‘Hongzi watermelon’ had resistance to *M. incognita*. The root knot diameter of ‘Hongzi watermelon’ was about half of that of ‘M16’ at DPI30, and the body width of nematodes in the root was only 26.8% of that of ‘M16’. In addition, the development of root knot was obviously slow, and only developed to the J3 and J4 stages of the life cycle, with little adults. However, at the same time, nematodes in the roots of ‘M16’ were mature and eggs were excreted, indicating that ‘Hongzi watermelon’ had a strong resistance to root-knot nematodes, which was similar to the research on ‘CM3’.

Phenolic compounds play an important role in resistance to plant abiotic stress, disease and insects, and their products include anthocyanins, tannins, flavonols and lignin. It was found that phenolic compounds were toxic to root-knot nematodes, and their content was positively correlated with plant nematode resistance [39,40,41]. Xu et al. [42] found that the total phenol content in the root of eggplant-resistant rootstock was higher than that of susceptible rootstock under no *M. incognita* infection, and the total phenol content further increased after *M. incognita* infection. In this study, the phenylpropanoid biosynthesis pathway was significantly enriched, and lignin was one of the main products of this pathway, which may play a role in the process of nematode infection. In general, when plants respond to the invasion of root-knot nematodes, they will inhibit the infection through physical defense, among which increasing lignin content and increasing cell wall thickness are the most important defense measures [43]. Studies on disease-resistant sweet potato and pickle cucumbers showed that the root cell wall of disease-resistant plants was thick, and the degree of lignification was high, so that nematodes could not further invade [44,45], and Bendezu [46] found similar results in the study of disease resistant peanuts. In our data, genes related to lignin biosynthesis (*Cla97C11G219500*, *Cla97C09G172390*) were significantly up-regulated, which may increase cell wall thickness and inhibit the physical penetration of nematode.

In addition, tannin is another kind of polyphenol compounds in plants, and the astringency of tannin is a self-defense mechanism for plants to resist insects and herbivores, which combines with digestive enzymes in the alimentary organ of insect to form complex, precipitate protein and inhibit enzyme activity, so as to reduce the digestion and utilization of nutrients and inhibit the development of insects [47,48,49,50,51]. Shen et al. [52] introduced *Apocynum venetum* DNA with high tannin content into cotton ovary and bred cotton germplasm with resistance to cotton bollworm and cotton aphid. The tannin content increased by 136.70% compared with the wild-type, significantly inhibiting the growth and development of newly hatched cotton bollworm. Dong et al. [50] found the number of aphids increased with the increase in inoculation days on wild-type plants, while the number increased slowly on transgenic plants with high tannin, and showed a downward trend in the later stage, indicating that the increase in tannin in alfalfa plays a role in aphid resistance or aphid avoidance. In this study, we identified one significantly up-regulated UDP glucoronosyl/UDP glucosyl transferase gene (*UGT84A13*, *Cla97C02G033370*). UGT84A13 is the first step reaction enzyme of gallotannin, which plays an important role in tannin synthesis [53]. In addition, we also found that the development of nematodes was significantly suppressed in ‘Hongzi watermelon’, indicating that it may inhibit the development of root-knot nematodes by increasing the content of tannin and thereby reducing the harm.

As one of the largest transcription factor families in plants, WRKY TF family members play an important role in regulating plant gene expression at the transcriptional level and responding to biotic and abiotic stresses [31]. Previous studies showed that WRKY TFs are closely related to the expression of nematode resistance genes in the incompatible and compatible interaction between root-knot nematodes and the host plant [54,55,56]. For example, Li et al. [31] showed that the *CaWRKY2* gene was induced by root-knot nematode and was closely related to the resistance gene of root-knot nematode in pepper. However, Chinnapandi et al. [57] showed that the high expression of *WRKY45* provided favorable conditions for nematode development in the root. In this study, one *WRKY* gene (*Cla97C02G027320*) was significantly up-regulated, indicating that it may play an important role in the resistance of the resistant variety to root-knot nematode infection. In addition, the MYB transcription factor also plays an important role in resistance to root knot nematode. In peach, MYB TF also regulates the expression of anthocyanins and other flavonoid compounds in the root of (*Prunus kansuensis* L.) through the phenylpropane metabolism pathway, so as to resist root-knot nematode infection [58]. Two significantly up-regulated MYB genes (*Cla97C08G148320* and *Cla97C01G012490*) were identified from our data, which were suspected to be involved in the protection against the infection of root-knot nematode. Furthermore, the expression of TF such as AP2/ERF is also closely related to the process of root knot formation after root-knot nematode infection. Warmerdam et al. [59] found that the abiotic stress tolerance mediated by ERF6 formed a new understanding of *Arabidopsis* sensitivity to *M. incognita*. In *Siraitia grosvenorii*, the expression of AP2/ERF TFs after infection was the most significant compared with that before infection with *M. incognita* [60]. In this study, an AP2/ERF gene (*Cla97C11G221160*) was significantly up-regulated, which may be involved in the resistance process of *M. incognita* infection.

At present, many studies have shown that SA, JA and ETH mediate the resistance of rice to *Meloidogyne graminicola* [19,61,62], whereas ABA, Brassinolide (BR) and monocrotalide (SLS) mediate the susceptibility of rice to *M. graminearum* and negatively regulate the resistance of rice [63,64,65,66]. In our transcriptome data, one JA-related gene (*Cla97C11G217710*) was significantly up-regulated, which may be related to nematode resistance. However, ETH-related genes (*Cla97C02G027510*, *Cla97C08G159750* and *Cla97C07G144030*) and ABA-related genes (*Cla97C05G099080* and *Cla97C10G186260*) were significantly down-regulated, which may be related to the early senescence of ‘M16′ because, in the middle and late stages of the development of ‘M16’, the continuous growth of nematode caused great mechanical damage to the root tissue. In addition, the nematode absorbed more nutrients, resulting in the shortage of plant nutrients and serious damage to the plant.

The production of reactive oxygen species (ROS) is likewise a manifestation of plant resistance to root-knot nematodes [67]. In response to nematode infection, plants activate a variety of oxidants and peroxidases, resulting in the production of ROS (superoxide anion radicals, singlet oxygen, hydrogen peroxide and hydroxy), which can poison the nematode and inhibit its infection process. For example, in tomato, increasing ROS content can reduce the infection rate of *M. incognita* [68]. In peanut, the ROS level was significantly increased after nematode infection, which may lead to resistance reaction or incompatibility reaction [69]. The seedling of the resistant tomato rootstock ‘Banzhen 2’ had potent antioxidant capacity and high ROS level and effectively inhibited the infection process of nematode, which could effectively inhibit the infection process of nematode [70]. In ginger, the leaves and roots accumulated a higher concentration of superoxide anion radicals and hydrogen peroxidefter during *M. incognita* infection [71].

The activity of SABP is similar to that of catalase, and the binding of SABP with SA can inhibit the activity of catalase, increase the level of hydrogen peroxide in plant cells and promote the production of SAR (salicylic-acid-mediated pathway of plant system acquiring resistance signal) [72,73]. In this study, one gene (*Cla97C01G015610*) that encodes SABP was significantly up-regulated, indicating that ROS scavenging system in ‘Hongzi watermelon’ was activated after *M. incognita* infection, which may be because it produced a large number of ROSs and finally effectively inhibited the infection. However, the expression level gradually decreased along with the increase in infection time. Meanwhile, we also identified one down-regulated *SABP* gene (*Cla97C09G179030*), indicating that the regulation process of SABP on ROS was a dynamic process, with the purpose to prevent excessive ROS from damaging plant tissues so as to trigger the ROS clearance system in time, which can effectively inhibit the development of nematodes without causing harm to the plant itself.

## 5. Conclusions

This study provides a transcriptome dataset for exploring the molecular mechanisms of watermelon resistance to *M.* *incognita* based on the resistant variety ‘Hongzi watermelon’ and the susceptible variety ‘M16’ watermelon. There were 3645, 2306, 4449 and 2362 DEGs identified in the four comparison groups (A0_B0, A1_B1, A2_B2 and A3_B3), and 835 shared DEGs among the four comparison groups were screened. A KEGG pathway enrichment analysis of the 835 DEGs showed that pathways of phenylpropane biosynthesis and carbon metabolism were significantly enriched. Furthermore, we analyzed the DEGs with the focus on discussing DEGs related to phenolic compounds, transcriptional factors, phytohormones and defense-related proteins, speculating that they play an important role in watermelon resistance to *M. incognita*. RNA-seq data of this study will contribute to a better understanding of the molecular mechanism of watermelon resistant to *M. incognita*.

## Data Availability

The datasets for this study can be found in the National Center for Biotechnology Information (NCBI) repository, bioproject: PRJNA675374.

## Supplementary Materials

The following supporting information can be downloaded at: https://www.mdpi.com/article/10.3390/life12071003/s1. Table S1: Primer sequences for qRT-PCR; Table S2: Summary of the quality assessment of reads and alignment statistics of RNA-seq mapped to reference genome; Table S3: KEGG pathway enrichment analysis; Table S4: Changes in DEGs related to transcriptional factors during *M. incognita* infection.

## Abbreviations

DEG	differential expression gene
ABA	abscisic acid
SA	salicylic acid
GA	gibberellin
FDR	false discovery rate
ROS	reactive oxygen species
BP	biological process
MF	molecular function
CC	cell component
CPC	coding potential calculato
ORF	open reading frame
FPKM	fragments per kilobase of transcript per million fragments
qRT-PCR	quantitative real time PCR
ABC transporters	ATP binding cassette transporters
LTPs	lipid transfer proteins
SABP	salicylic acid-binding protein
4CL	4-coumaric acid-CoA ligase
C3H	coumaric acid 3-hydroxylase
CSE	caffeoyl shikimate esterase
COMT	caffeic acid-O-methyltransferase
CCR	cinnamyl CoA reductase
PRX	peroxidase
DPI	days post-infection
TF	transcription factor

## References

[1] Krishnaveni M., Subramanian S. (2005). Root-knot nematodes of cucurbits and their management—A review. Agric. Rev..

[2] Thies J.A., Buckner S., Horry M., Hassell R., Levi A. (2015). Influence of *Citrullus lanatus* var. *citroides* rootstocks and their F1 hybrids on yield and response to root-knot nematode, *Meloidogyne incognita*, in grafted watermelon. Hortscience.

[3] Netscher C., Sikora R. (1990). Nematode Parasites of Vegetables.

[4] Lu Z., Chen M., Hu X., Huang J., Xi X., Wang Y. A study on the sensitivity of main vegetables produced in Beijing greenhouse to root knot nematode. Proceedings of the 13th National Symposium on Plant Nematodes.

[5] Zhao H., Peng D.L., Zhu J.L. (2003). Reviews on the root-knot nematodes. Plant Prot..

[6] Zhang X.W., Qian X.L., Liu J.W. (1989). Evaluation on the resistance to root-knot nematode of watermelon germplasm and control for it. J. Fruit Sci..

[7] Vela M.D., Giné A., López-Gómez M., Sorribas F.J., Ornat C., Verdejo-Lucas S., Talavera M. (2014). Thermal time requirements of root-knot nematodes on zucchini-squash and population dynamics with associated yield losses on spring and autumn cropping cycles. Eur. J. Plant. Pathol..

[8] Verdejo-Lucasa S., Talavera M. (2019). Root-knot nematodes on zucchini (*Cucurbita pepo* subsp. pepo): Pathogenicity and management. Crop. Prot..

[9] Shahbaz M.U., Mukhtar T., Irfanulhaque M., Begum N. (2015). Biochemical and serological characterization of *Ralstonia solanacearum* associated with chilli seeds from Pakistan. Int. J. Agric. Biol..

[10] Fu L. (2015). Preliminary study on the occurrence and control technology of plant pathogenic nematode. Agric. Technol..

[11] Morris K.A., Langston D.B., Davis R.F., Noe J., Dickson D., Timper P. (2016). Efficacy of various application methods of fluensulfone for managing root-knot nematodes in vegetables. J. Nematol..

[12] Thies J.A., Levi A. (2007). Characterization of watermelon (*Citrullus lanatus* var. *citroides*) germplasm for resistance to root-knot nematodes. Hortscience.

[13] Shen Z., Li X., Feng L., Wang H., Song J., Yang C., Gong H. (2007). Evaluation on resistance of *Cucurbitaceae* germplasm resources to root-knot nematode. J. Plant Genet. Resour..

[14] Thies J.A., Levi A., Ariss J.J., Hassell R. (2015). RKVL-318, a root-knot nematode-resistant watermelon line as rootstock for grafted watermelon. Hortscience.

[15] Deng Y., Wang Z., Sun D., Lu J., Ma S., Zhu Y., Liu J. (2012). Screening and evaluation of watermelon germplasm for resistance to *Meloidogyne incognita*. J. Fruit Sci..

[16] Punithaveni V., Jansirani P., Sivakumar M. (2015). Screening of cucurbitaceous rootstocks and cucumber scions for root knot nematode resistance (*Meloidogyne incognita* Kofoid and White). Electron. J. Plant Breed..

[17] Wang Z., Gerstein M., Snyder M. (2009). RNA-Seq: A revolutionary tool for transcriptomics. Nat. Rev. Genet..

[18] Tang H.G., Huang Y.H., Lu H.B., Tian D.Y., Zhang Y.W. (2016). The application perspective of transcriptome sequencing on discovering the genes and variety breeding of bioenergy grass. Acta Agrestia Sin..

[19] Kyndt T., Denil S., Haegeman A., Geert Trooskens G., Bauters L., Criekinge W.V., Meyer T.D., Gheysen G. (2012). Transcriptional reprogramming by root knot and migratory nematode infection in rice. New Phytol..

[20] Olga A., Maria H., Jonathan S., Andrea S., Lev G. (2015). Transcriptome analysis of resistant and susceptible alfalfa cultivars infected with root-knot nematode *Meloidogyne incognita*. PLoS ONE.

[21] Xue Y.Y., Deng Y., Liu J.P., Xu X.J., Zhu Y.C., An G.L., Li W.H., Sun D.X. (2015). Molecular cloning and expression analysis of root-knot nematode resistance related aene in watermelon. J. Fruit Sci..

[22] Li K. (2016). Screening of Watermelon and Melon Rootstock Resources Resistant to *Meloidogyne Incognita* and Transcriptomic Analysis. Master’s Thesis.

[23] Ling J., Mao Z., Zhai M., Zeng F., Yang Y.H., Xie B.Y. (2017). Transariptome profiling of *Cucumis metuliferus* infected by *Meloidogyne incognita* provides new insights into putative defense regulatory network in *Cucurbitaceae*. Sci. Rep..

[24] Li S. (2018). Study on Transcriptome and Protein Interaction Network of “Huipizhi Heidou” Resistance to Soybean Cyst Nematode. Ph.D. Thesis.

[25] Wang X.W. (2013). Identification of Root-Knot Nematodesfrom Protected Cultivation and Resistance of Vegetable Cultivars. Master’s Thesis.

[26] Guo S.G., Zhao S.J., Sun H., Wang X., Wu S., Lin T., Ren Y., Gao L., Deng Y., Zhang J. (2019). Resequencing of 414 cultivated and wild watermelon accessions identifies selection for fruit quality traits. Nat. Genet..

[27] Trapnell C., Roberts A., Goff L., Pertea G., Kim D., Kelley D. (2014). Differential gene and transcript expression analysis of RNA-seq experiments with TopHat and Cufflinks. Nat. Protoc..

[28] Yuan G.P., Liu J.P., An G.L., Li W.H., Si W.J., Sun D.X., Zhu Y. (2021). Genome-wide identification and characterization of the treha-lose-6-phosphate synthetase (*TPS*) gene family in watermelon (*Citrullus lanatus*) and their transcriptional responses to salt stress. Int. J. Mol. Sci..

[29] Livak K.J., Schmittgen T.D. (2001). Analysis of relative gene expression data using real-time quantitative PCR and the 2^−∆∆CT^ method. Methods.

[30] Chen C.J., Chen H., Zhang Y., Thomas H., Frank M., He Y., Xia R. (2020). TBtools: An integrative toolkit developed for interactive analyses of big biological data. Mol. Plant.

[31] Li H., Li J., Cheng Y., Wei W., Li L., Chen Y. (2020). Identification and analysis of WRKY transcription factors in *Siraitia grosvenorii* under infection of *Meloidogyne incognita*. Plant Protoc..

[32] He S., Yuan G., Bian S., Han X., Liu K., Cong P., Zhang C. (2020). Major latex protein MdMLP423 negatively regulates defense against fungal infections in apple. Int. J. Mol. Sci..

[33] Chong J., Baltz R., Schmitt C., Beffa R., Fritig B., Sain-Drenan P. (2002). Downregulation of a pathogen-responsive tobacco UDP-Glc: Phenylpropanold glucosyltran sferase reduces scopoletin glucoside accumulation, enhances oxidative stress, and weakens virus resistance. Plant Cell.

[34] Gachon C., Baltz R., Saindrenan P. (2004). Over-expression of a scopoletin glucosyltransferase in *Nicotiana tabacum* leads to precocious lesion formation during the hypersensitive response to tobacco mosaic virus but does not affect virus resistance. Plant Mol. Biol..

[35] Sepulveda-Jimenez G., Rueda-Benitez P., Porta H., Ro-cha-Sosa M. (2005). A red beet (*Beta vulgaris*) UDP-glucosyltrans-ferase gene induced by wounding, bacterial infiltration and oxidative stress. J. Exp. Bot..

[36] Ma J., Mao Z., Li H., Xie B. (2014). Resistance identification of *Cucumis metuliferus* to *Meloidogyne incognita* and characteristic analysis. Acta Hortic. Sin..

[37] Wei C., Shi Q.Q., Ma Y.Q., Ma J.H., Mao Z.C., Ling J., Yang Y.H., Xie B.Y. (2016). Effects of peroxidase gene to the resistance of *Cucumis metuliferus* against *Meloidogyne incognita* in different temperature. Acta Hortic. Sin..

[38] Wang Z. (2011). The Screening and Evaluation of Watermelon Rootstock Resources for Resistance to Meloidogyne Incognita. Master’s Thesis.

[39] Zhang Z. (2015). Phenolic Compositions and Antioxidant Capacity of the Fruits of Chinese Local Pummelos. Master’s Thesis.

[40] Dou L. (2019). The Negative Regulational Molecular Mechanism of the Poplar Transcription Factor MYB93 Involved in Flavonoids and Lignin Biosynthesis. Master’s Thesis.

[41] Fu M., Chen J., Xiao T., Rui K. (2008). Advances in studies on interaction mechanism between root-knot nematodes and host plants. Chin. J. Trop. Agric..

[42] Xu X. (2008). Study on Evaluation for Resistance to Meloidogyne Incognita and the Resistant Mechanism of Eggplant Rootstocks. Master’s Thesis.

[43] Zhao R.Z. (2018). Studies on the Transcriptional Analysis of Resistance Response of Watermelon to Meloidogyne Incognita and Induced Control by Red-Light. Master’s Thesis.

[44] Lin M., He L., Wen L., Fang Z. (1996). Mechanism of morphological structure of sweet potato resistance to potato rot nematode (*Ditylenchus destructor*). Sci. Agric. Sin..

[45] Ye D.Y., Wang X., Zhang Y.X., Qian C., Chen J.F. (2010). Anatomy and cytology of sour cucumber for its resistance to the root-knot nematode *Meloidogyne incognita*. Acta Phytopathol. Sin..

[46] Bendezu I.F., Starr J.L. (2003). Mechanism of resistance to *Meloidogyne arenaria* in the peanut cultivar COAN. J. Nematol..

[47] Li X., Wang W., Wu Y. (2005). Physiological function and economic value of plant tannin. J. West China For. Sci..

[48] Chen R., Gao H., Zhang G., Zhu K., Cheng W. (2020). Effects of secondary metabolites in wheat kernels on activities of three detoxifying enzymes and related gene expression in *Sitodiplosis mosellena*. Sci. Agric. Sin..

[49] Wu Y.Q., Guo Y.Y. (2001). Potential resistance of tannins-flavonoids in upland cottonagainst *Helicpverpa armigera*. Acta Ecol. Sin..

[50] Dong W., Chen C., Ma H. (2019). Analysis of insect resistance and herbicide resistance in transgenic alfalfa plants over-expressing the *OvBAN/bar* gene. Acta Prataculturae Sin..

[51] Lees G.L., Hinks C.F., Suttill N.H. (1992). Condensed tannins in some forage legumes their rolein the improvement of ruminant pasture bloat. Basic Life Sci..

[52] Shen F., Yu Y.J., Zhang X., Liu F., Yin C. (1999). Insect resistant stock selected byintroducing dogbane DNA into cotton. Acta Agric. Boreali-Sin..

[53] Mittasch J., Böttcher C., Frolova N., Bönn M., Milkowski C. (2014). Identification of UGT84A13 as a candidate enzyme for the first committed step of gallotannin biosynthesis in pedunculate oak (*Quercus robur*). Phytochemistry.

[54] Jammes F., Lecomte P., de Almeida E.J., Bitton F., Martin-Magniette M., Renou J., Abad P., Favery B. (2005). Genome-wide expression profiling of the host response to root-knot nematode infection in *Arabidopsis*. Plant J..

[55] Li S., Mao Z., Li L., Feng D., Yang Y., Xie B. (2008). Isolation of *WRKY* genes in the incompatible interaction between *Meloidogyne incognita* and capsicum annuum. Acta Hortic. Sin..

[56] Zheng J. (2011). Isolation, Expression and Function Analysis of the WRKY Transcription Factor Gene CaWRKY6 and CaWRKY30 in Pepper. Ph.D. Thesis.

[57] Chinnapandi B., Bucki P., Miyara S.B. (2017). *SIWRKY45*, nematode-responsive tomato *WRKY* gene enhances susceptibility to the *M. javanica* infection. Plant Signal. Behav..

[58] Wei X. (2010). Cloning and Localization of Root-Knot Nematode Resistance Relative MYB Transcription Factor Genes in ‘Honggengansutao Peach’ (*Prunus kansuensis* L.). Master’s Thesis.

[59] Warmcrdam S., Sterken M.G., Schaik C.V., Oortwijn M.E.P., Lozano-Torre J.L., Bakker J., Goverse A., Smant G. (2019). Mediator of tolerance to abiotic stress ERF6 regulates susceptibility of *Arabidopsis* to *Meloidogyne incognita*. Plant Pathol..

[60] Li L. (2019). Transcriptomic and metabolomics study of the early interactions between *Siraitia grosvenorii* roots and *Meloidogyne incognita*. Master’s Thesis.

[61] Nahar K., Kyndt T., de Vleesschauwer D., Hofte M., Gheysen G. (2011). The jasmonate pathway is a key player in systemically induced defense against root-knot nematodes in rice. Plant Physiol..

[62] Verbeek R., van Buyten E., Alam M., de Vleesschauwer D., van Bockhaven J., Asano T., Kikuchi S., Haeck A. (2019). Jasmonate-induced defense mechanisms in the belowground antagonistic interaction between *Pythium arrhenomanes* and *Meloidogyne graminicola* in rice. Front. Plant Sci..

[63] Nahar K., Kyndt T., Nzogela Y.B., Gheysen G. (2012). Abscisic acid interacts antagonistically with classical defense pathways in rice-migratory nematode interaction. New Phytol..

[64] Nahar K., Kyndt T., Hause B., Hofte M., Godelieve G. (2013). Brassinosteroids suppress rice defense against root-knot nematodes through antagonism with the jasmonate pathway. Mol. Plant Microbe Interact..

[65] Yimer H., Nahar K., Kyndt T., Haeck A., van Meulebroek L., Vanhaecke L., Demeestere K., Hofte M., Gheysen G. (2018). Gibberellin antagonizes jasmonate-induced defense against *Meloidogyne graminicola* in rice. New Phytol..

[66] Godelieve G., Melissa G. (2018). Phytoparasitic nematode control of plant hormone pathways. Plant Physiol..

[67] Baker E., Dees R., Bakker J. (2005). Mechanisms Involved in Plant Resistance to Nematodes.

[68] Vos C., Schouteden N., Tuinen D.V. (2013). Mycorrhiza-induced resistance against the root-knot nematode *Meloidogyne incognita* involves priming of defense gene responses in tomato. Soil Biol. Biochem..

[69] Holajjer P., Chakraborty K., Nataraja M.V. (2016). Differential reactive oxygen species modulation in leaves of groundnut due to root-knot nematodes, *Meloido gnearenaria* and *M. incognita*. J. Nematol..

[70] Jia S. (2012). Study on Evaluation and Mechanism of Tomato Rootstocks for Resistance to Meloidogyne Incognita. Ph.D. Thesis.

[71] Guo Y., Wang X., Xu K., Zhang G. (2005). Effect of *Meloidogyne incognita* on the physiological and chemical changes in ginger. Acta Phytopathol. Sin..

[72] Park S., Liu P., Forouhar F., Vlot A., Tong L., Tietjen K., Klessig D. (2009). Use of a synthetic salicylic acid analog to investigate the roles of methyl salicylate and its esterases in plant disease resistance. J. Biol. Chem..

[73] Forouhar F., Yang Y., Kumar D., Chen Y., Fridman E., Park S., Chiang Y., Acton T., Montelione G., Pichersky E. (2005). Structural and biochemical studies identify tobacco SABP2 as a methyl salicylate esterase and implicate it in plant innate immunity. Proc. Natl. Acad. Sci. USA.

